# Quality of Life and Late Complications After Minimally Invasive Compared to Open Esophagectomy: Results of a Randomized Trial

**DOI:** 10.1007/s00268-015-3100-y

**Published:** 2015-06-03

**Authors:** K. W. Maas, M. A. Cuesta, M. I. van Berge Henegouwen, J. Roig, L. Bonavina, C. Rosman, S. S. Gisbertz, S. S. A. Y. Biere, D. L. van der Peet

**Affiliations:** Department of Surgery, VU University Medical Center, Amsterdam, The Netherlands; Department of Surgery, Academic Medical Center, Amsterdam, The Netherlands; Department of Surgery, Hospital Universitari de Girona Dr Josep Trueta, Griona, Spain; Department of Surgery, Istituto de Ricovero e Cura a Carattere Scientifico Policlinico San Donato, University of Milan, Milan, Italy; Department of Surgery, Canisius Wilhelmina Hospital, Nijmegen, The Netherlands

## Abstract

**Background:**

The minimally invasive esophagectomy (MIE) is widely being implemented for esophageal cancer in order to reduce morbidity and improve quality of life. Non-randomized studies investigating the mid-term quality of life after MIE show conflicting results at 1-year follow-up. Therefore, the aim of this study is to determine whether MIE has a continuing better mid-term 1-year quality of life than open esophagectomy (OE) indicating both a faster recovery and less procedure-related symptoms.

**Methods:**

A one-year follow-up analysis of the quality of life was conducted for patients participating in the randomized trial in which MIE was compared with OE. Late complications as symptomatic stenosis of anastomosis are also reported.

**Results:**

Quality of life at 1 year was better in the MIE group than in the OE group for the physical component summary SF36 [50 (6; 48–53) versus 45 (9; 42–48) *p* .003]; global health C30 [79 (10; 76–83) versus 67 (21; 60–75) *p* .004]; and pain OES18 module [6 (9; 2–8) versus 16 (16; 10–22) *p* .001], respectively. Twenty six patients (44 %) in the MIE and 22 patients (39 %) in the OE group were diagnosed and treated for symptomatic stenosis of the anastomosis.

**Conclusions:**

This first randomized trial shows that MIE is associated with a better mid-term one-year quality of life compared to OE.

## Introduction

Esophagectomy with lymphadenectomy after neoadjuvant chemoradiotherapy or chemotherapy is regarded as the only curative option for patients with resectable esophageal cancer [1–3]. This operative procedure has a high incidence of postoperative complications, especially pulmonary infections and is also associated with an impaired quality of life [4]. Minimally invasive procedures are increasingly implemented for reducing such complications and improving postoperative quality of life.

To date, the short-term results of only one randomized trial have been published. This multicenter, randomized trial provides evidence for certain short-term benefits of the minimally invasive approach for patients with resectable esophageal cancer. It reported significantly less pulmonary infections after the minimally invasive esophagectomy (MIE) as well as better pain scores, less blood loss and a shorter hospital stay. Importantly, this trial showed a better short-term quality of life at 6 weeks after surgery for the patients who underwent a MIE procedure [5].

The quality of life after open transthoracic esophagectomy usually improves within 1 year [6]. However, studies investigating the mid-term quality of life after MIE show conflicting results at 1-year follow-up [7–13]. Furthermore, these results are based on analysis of patient series and non-randomized study design.

Therefore, a 1-year follow-up analysis of the quality of life was conducted for patients participating in the randomized trial in which MIE was compared with open esophagectomy (OE) [5, 14]. We investigated if MIE has a continuing better mid-term 1 year quality of life than OE indicating both a faster recovery and less procedure-related symptoms. Additionally, late complications and 1-year follow-up survival data are also reported.

## Methods

### Study design and patients

This study at 1-year follow-up is an analysis of a multicenter, randomized trial which was performed between June 1, 2009 and March 31, 2011 at five centers: two in Amsterdam (Netherlands), and one in Nijmegen (Netherlands), Girona (Spain), and Milan (Italy) [5]. Eligible participants had resectable esophageal cancer (cT1–3,N0–1, M0), histologically proven adenocarcinoma, squamous cell carcinoma, or undifferentiated carcinoma of the intrathoracic esophagus and gastro-esophageal junction. Patients were aged 18–75 years and had a WHO performance status of two or less. We excluded patients with cervical esophageal cancer or another malignancy.

For quality assurance, the principal investigator visited all centers interested in trial participation. Minimally invasive esophagectomies were observed in person by the principal investigator. Both procedures were done by surgeons experienced in open esophageal resection, and with extensive experience in minimally invasive procedures, who had done at least ten MIE. Only hospitals with more than 30 esophagectomies per year participated and their medical ethics boards approved the trial. Diagnosis and staging was established before neoadjuvant treatment by esophagoscopy and biopsies; CT scans of the neck, thorax, and abdomen; and endo-ultrasonography.

Surgeons at the outpatient clinic informed eligible patients of the treatment regimen. Written informed consent was obtained from included patients. We used a computer-generated randomisation sequence to randomly assign patients, in a 1:1 ratio, to undergo either open or minimally invasive esophagectomy. Randomisation was stratified by study center. All participating centers compiled an exclusion list to analyse the quality of the randomisation rate. Patients, and investigators undertaking interventions, assessing outcomes, and analysing data were not masked to group assignment.

### Operative procedure

Patients in both groups received identical pre-and postoperative treatment. For most patients, neoadjuvant treatment consisted of weekly administrations of 50 mg/m2 paclitaxel plus carboplatin (Calvert’s formula for dosing; area under the concentration–time curve 2 for 5 weeks) and concurrent radiotherapy (41,4 Gy in 23 fractions for 5 days per week). After 6–8 weeks, neoadjuvant treatment was followed by surgery by open or minimally invasive esophagectomy.

Open esophagectomy involved a right posterolateral thoracotomy in the lateral decubitus position with double tracheal intubation and lung block, midline laparotomy, and cervical or intrathoracic anastomosis. MIE was performed through a right thoracoscopy in the prone position with single-lumen tracheal intubation, upper abdominal laparoscopy, and cervical incision. For patients undergoing MIE with an intrathoracic anastomosis, a bronchus blocker was placed in the right bronchus to help with one-lung ventilation during anastomosis.

Both procedures included a two-field esophageal resection with 3–4 cm wide gastric tube formation followed by a cervical or intrathoracic anastomosis. Further details of the surgical techniques for open and MIE have been published elsewhere [15].

In the first 3 days after surgery, patients received epidural analgesia. If epidural analgesia was unsuccessful, patient-controlled analgesia with intravenous opioids was given. Enteral feeding was started on day 1 after surgery through a percutaneous jejunostomy catheter.

### Study endpoints

The primary short-term endpoint of the study was postoperative pulmonary infection, defined as clinical manifestation of pneumonia or bronchopneumonia confirmed by thoracic radiographs or CT scan (assessed by independent radiologists) and a positive sputum culture, within the first 2 weeks of surgery and during the whole stay in hospital.

Secondary short-term endpoints included among others postoperative complications other than pulmonary infections (e.g., anastomotic leakage, vocal cord paralysis confirmed by laryngoscopy), quality of life [assessed by short form 36 (SF 36) Health Survey (version 2) and European Organization for Research and Treatment of Cancer (EORTC) quality of life questionnaires C30 and OES18 module].

Mid-term secondary endpoints included quality of life at 1 year (assessed by SF 36 and EORTC C30 and OES18 module), incidence of late complications (e.g., anastomotic stenosis) and overall and disease-free survival. Data were collected prospectively during outpatient visits by paper questionnaires. Radiological and/or endoscopic assessment (for recurrence or metastasis) was performed only by indication if the history and physical examination of the patient lead to suspicion.

### Statistical analysis

We used Power and Precision (version 2) for sample size calculation for the short-term study. Previous data indicated a 28 % difference in pulmonary infections between minimally invasive (29 %) [7, 15–18] and open (57 %) esophagectomy [19]. To show a difference of this magnitude, two groups of 48 patients would be needed (*α* 0.05, *β* 0.80). With an estimation that about 20 % of the eligible patients might not undergo the allocated intervention (e.g., due to metastases during neoadjuvant treatment or unresectable tumors), we enrolled 60 patients per group. We expressed data as median and range for continuous variables, or mean and SDs when appropriate. We expressed distributions of dichotomous data in percentages. When appropriate, we compared groups with an independent samples t test, otherwise a Mann–Whitney *U* test, or *χ*^2^ test. We calculated relative risk (RR) for the primary endpoint with 95 % CIs. Data were analysed according to the intention-to-treat principle. We did statistical analysis with SPSS (version 17).

## Results

### Subjects

**Fig. 1:**
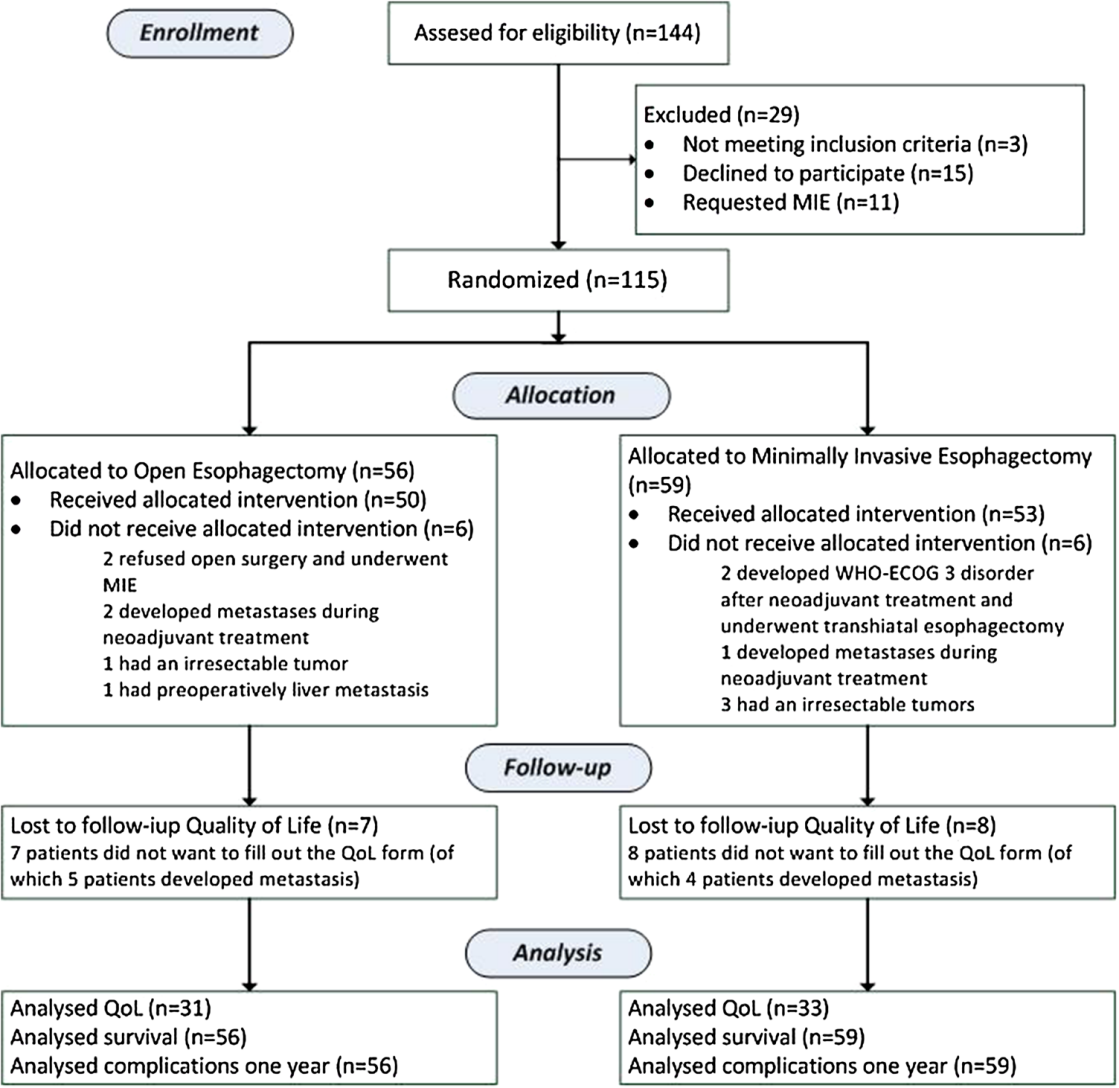
Trial profile *MIE* minimally invasive esophagectomy, *WHO-ECOG* World Health Organization- Eastern Cooperative Oncology Group, *QoL* quality of life. Analysis: intention-to-treat

We randomly assigned 115 of 144 eligible patients to receive either OE or MIE. Four crossovers occurred: two patients assigned to the OE group underwent MIE, and two assigned to MIE developed a WHO-ECOG score of 3 during neoadjuvant treatment and were treated by transhiatal esophagectomy. Eight patients did not undergo a resection (Fig. 1); we included these patients in the analysis of the allocated group according to the intention-to-treat principle. Fifty-six patients were analysed in the OE group and 59 in the MIE group. The demographic and clinical characteristics of the two groups were similar at baseline (Table 1). The short-term results are reported elsewhere [5].

**Table 1:** Baseline demographic and clinical characteristics of the intention-to-treat population

	OE (*N* = 56)	MIE (*N*-59)
Gender		
Male	46 (82 %)	43 (73 %)
Female	10 (18 %)	16 (27 %)
Age (years)*	62 (42–75)	62 (34–75)
BMI (kg/m^2^)†	24 (3.7)	25 (3.6)
ASA classification		
1	15 (27 %)	10 (17 %)
2	32 (57 %)	34 (58 %)
3	8 (14 %)	14 (24 %)
4	1 (2 %)	1 (2 %)
Type of carcinoma		
Adenocarcinoma	36 (64 %)	35 (59 %)
Squamous cell	19 (34 %)	24 (41 %)
carcinoma	1 (2 %)	0 (0 %)
Other neoadjuvant treatment		
Chemoradiotherapy	52 (93 %)	54 (92 %)
Chemotherapy alone	4 (7 %)	5 (8 %)
Location of tumor††		
Upper third	3 (5 %)	1 (2 %)
Middle third	22 (39 %)	26 (44 %)
Lower third or gastro-esophageal junction	31 (55 %)	32 (54 %)
Level of anastomosis		
Cervical	37 (66 %)	38 (64 %)
Thoracic	15 (27 %)	17 (29 %)
Total lymph nodes retrieved*		
Resection margin¶	21 (7–47)	20 (3–44)
R0	47 (84 %)	54 (92 %)
R1	5 (9 %)	1 (2 %)
pStage§		
0	0 (0 %)	1 (2 %)
I	4 (7 %)	4 (7 %)
IIa	16 (29 %)	17 (29 %)
IIb	6 (11 %)	9 (15 %)
III	14 (25 %)	11 (19 %)
IV	5 (9 %)	4 (7 %)
No residual tumor of lymph-node metastasis	7 (13 %)	9 (15 %)

Data are *n* (%), median (range), and mean (SD)

*OE* open esophagectomy, *MIE* minimally invasive esophagectomy, *BMI* body-mass index, *ASA* American Association of Anesthesiologists

* Skewed distribution, Mann–Whitney test applied

† Normal distribution, independent samples *t* test applied

†† American Joint Committee on cancer site classification of thoracic and abdominal esophagus

¶ efined as >1 mm from a resection marge

§ Staging based on the American Joint Committee on cancer, 6th edn; four patients in each group did not undergo resection due to metastasis or irresectability

### Quality of life

Quality of life questionnaires were obtained at 1 year postoperatively. A response compliance of 82 % by patients was obtained. Under the non-responders were nine patients with recurrence; these patients were equally distributed between both groups. Moreover, six patients did not complete the questionnaires for unknown reasons, also equally distributed between both groups.

Quality of life questionnaire results are shown in Table 2. Overall the quality of life scores at 1 year were better for both groups compared at 6 weeks and preoperatively when using the SF-36 and EORTC C-30 Global health domain and the EORTC OES-18 domains. Importantly, there are significantly better scores after 1-year follow-up for the MIE group as compared to the OE group. These differences are present in three domains: physical activity [SF36: 50 (6; 48–53) vs .45 (9; 42–48) *p* .003]; global health [C30: 79 (10; 76–83) vs. 67 (21; 60–75) *p* .004]; and pain [OES18: 6 (9; 2–8) versus 16 (16; 10–22) *p* .001].

**Table 2:** Quality of life domains

	OE ( 31)	MIE (33)	*p* value
SF 36†			
Mental component summary			
Preoperatively	45 (9; 43-48)	46 (12; 43–49)	.955
6 weeks	45 (11; 40–50)	46 (10; 41–50)	.806
1 year	50 (10; 47–53)	53 (10; 49–56)	.317
Physical component summary			
Preoperatively	43 (9; 40–46)	46 (8; 44–48)	.072
6 weeks	36 (6; 34–39)	42 (8; 39–46)	.007
1 year	45 (9; 42–48)	50 (6; 48–53)	**.003**
EORTC C30†			
Global health			
Preoperatively	63 (23; 56–70)	66 (22; 60–72)	.631
6 weeks	51 (21; 44–58)	61 (18; 56–67)	.020
1 year	67 (21; 60–75)	79 (10; 76–83)	**.042**
EORTC OES 18‡			
Pain			
Preoperatively	23 (17–22, 22–30)	17 (24; 11–24)	.187
6 weeks	19 (13–21, 21–26)	8 (11; 5–11)	.002
1 year	16 (16; 10–22)	6 (9; 3–10)	**.003**
Talking			
Preoperatively	12 (25; 4–19)	10 (23; 4–17)	.745
6 weeks	37 (39; 25–49)	18 (26; 10–26)	.008
1 year	10 (21; 3–18)	5 (14; 0–11)	.288

Only the one-year QOL differences that were significant are in bold

Data are mean (SD, 95 %CI)

*OE* open esophagectomy, *MIE* minimally invasive esophagectomy, *EORTC* European Organization for Research and Treatment of Cancer Quality of Life Questionnaires, *SF* 36 Short Form 36 Health Survey (version 2)

† Measures general aspects of health; scores range from 0 to 100, with higher scores representing better well-being

‡ Assesses several aspects of esophageal function; scores range from 0 to 100, with lower scores indicating better function

Compared to postoperative levels, we see that after 1 year the patients in both groups improved their scores without significant differences of improvement between the groups (Table 3).

**Table 3:** Improvement (delta) of Quality of life in time

	OE	MIE	*p* value
SF36			
Mental component summary	10 (0.499)	10 (0.514)	0.546
Physical component summary	10 (0.327)	9 (0.406)	0.465
EORTC C30			
Global health	14 (0.518)	16 (0.498)	0.080
EORTC OES18			
Pain	−7 (0.366)	0 (−0.041)	0.065
Talking	−28 (0.256)	−18 (−0.083)	0.091

Data are mean difference between 6 weeks postoperatively and one year postoperatively (correlation coefficient)

*OE* open esophagectomy, *MIE* minimally invasive esophagectomy, *EORTC* European Organization for Research and Treatment of Cancer Quality of Life Questionnaires, *SF* 36 short form 36 health survey (version 2)

### Late complications

Late complications observed during the first year are depicted in Table 4.

**Table 4:** Late complications one year postoperatively

	OE (56)	MIE (59)	*p* value
Stenosis anastomosis	22 (39 %)	26 (44 %)	0.603
Intrathoracic herniation	1 (2 %)	0 (0 %)	0.303
Vocal cord paralysis	4 (7 %)	1 (2 %)	0.152

Data are *n* (%)

*OE* open esophagectomy, *MIE* minimally invasive esophagectomy

After 1 year, 26 patients (44 %) in the MIE and 22 patients (39 %) in the OE group were diagnosed and treated for symptomatic stenosis of the anastomosis. Endoscopic dilatations were performed with a median number of five dilatations [2–20]. One patient in the MIE group was re-admitted at 2 months postoperatively because of herniation of colon and small bowel in the thoracic cavity, necessitating reposition of viable bowels through median laparotomy.

Six weeks postoperatively, eight patients in the OE group and one patient in the MIO group suffered from unilateral vocal cord paralysis. One patient in the OE group had bilateral vocal cord paralysis versus none in the MIE group. After 1 year, three patients in the OE group recovered from the vocal cord paralysis versus 0 (none) in the MIE group. The patient with bilateral vocal cord paralysis regained function of one of the vocal cords; consequently his tracheotomy could be closed.

### Recurrence, distant metastasis, and survival

Data about local recurrence and distant metastases are shown in Table 5. Thirty-two patients died during the first year, 18 (32 %) in the OE group and 14 (23 %) in the MIE group (*p* = 0.314). Death was related principally to distant metastases (19 patients), without significant differences between the two groups (*p* = 0.167). Local recurrence was observed in three patients in the OE group (*p* = 0.072).

**Table 5:** Overall and disease-free survival at one year

	OE (56)	MIE (59)	*p* value
Overall survival	38 (68 %)	45 (76 %)	0.314
Disease-free survival	33 (59 %)	41 (69 %)	0.237
Causes of death	18 (32 %)	14 (24 %)	0.314
Metastases	12 (21 %)	7 (12 %)	0.167
Recurrence	3 (5 %)	0 (0 %)	0.072
In hospital mortality	1 (2 %)	2 (3 %)	0.590
Irresectable tumor	1 (2 %)	3 (5 %)	0.335
Other causes	1 (2 %)	2 (3 %)	0.590

Data are *n* (%)

*OE* open esophagectomy, *MIE* minimally invasive esophagectomy

## Discussion

In this trial, MIE resulted in a better mid-term 1-year quality of life for the physical component summary of the SF-36 questionnaire, EORTC C30 global health domain and OES 18 pain domain compared to open esophagectomy. In addition, there were no differences in survival and late complications at 1 year between the groups.

The impact of the surgical procedure is apparently of influence even after 1 year. The better physical domains of the SF-36 and pain of the OES 18 module for the MIE group indicate fewer limitations for the patients probably due to less surgical trauma by the smaller incisions which are used for MIE. Post-thoracotomy-related pain is well known and widely reported [20, 21]. It is probably a combination of intercostal nerve damage and myofascial pain [22]. It has been reported that up to 50 % of patients describe post-thoracotomy pain 1 year after the procedure [23]. A recent study on the type of thoracotomy found an inverse relationship between the incision length and post-thoracotomy pain [24]. With MIE both incision length and myofascial damage is limited. This could explain the better outcome at 1 year after MIE. In addition, the abdominal incision is also smaller in MIE compared to OE. However, the influence of abdominal wall pain after laparotomy at 1 year is probably marginal compared to the influence of post-thoracotomy pain on quality of life. Therefore, we found the difference specifically in the chest pain domain of the EORTC OES18 and it did not appear in the overall bodily pain domain of the SF-36. The experience of pain and dysfunction in the right shoulder and protracted pain in the thoracotomy scar is frequent and relevant as these effects were observable in the clinical differences at the outpatient clinic. As the scores in the OES-18 have shown, the post-thoracotomy pain syndrome had a negative influence upon daily activities in one third of patients.

Postoperative health-related quality of life is impaired in patients with esophageal cancer in comparison to preoperative levels [8, 9] and to that of healthy reference populations [25–27]. The recovery after esophagectomy to preoperative levels was present for both groups at 1 year. Quality of life data after MIE is limited. Parameswaran et al. studied 97 patients undergoing OE, laparoscopic-assisted esophagectomy (LAE), or MIE for high-grade dysplasia or cancer [9]. Patients completed validated questionnaires before, after 6 weeks, at three and 6 months after surgery. Following surgery, the fatigue levels increased dramatically and activity levels reduced in all groups after 6 weeks. These gradually recovered to baseline levels following MIE and LAE within 6 months, but the scores regarding ability to perform activities of daily living and most indicators of fatigue had not returned to baseline levels in the OE group. Although the studied questionnaires in the above mentioned study differs from our protocol and is of non-randomized design, nonetheless it is clear that the trend is comparable. In our study, we also see a drop at 6 weeks after surgery and even better results than baseline after 1 year. At 6 weeks postoperatively it was clear that the difference between the groups was clinically significant as the minimally invasive group had significant fewer pulmonary complications, probably due to other factors such as less surgical trauma and less pain [5]. At 1 year the same differences were present and thus also clinically significant. In our opinion, all differences between the groups in the specific domains result in a clinically important difference, which is best understood for the pain domain due to post-thoracotomy pain. The improvement of quality of life after 1 year was equal for both groups compared to 6 weeks postoperatively.

Furthermore, in this trial, overall survival and disease-free survival rates after 1 year were not different between the groups. This data are in concordance with recent literature of patients treated by neoadjuvant therapy followed by esophageal resection [2, 3]. Local recurrence and distal metastases were also not different between the groups and comparable with other reports [3, 28]. However, reliable survival analysis is usually performed at 5 years postoperatively. This will be reported in the future.

The rate of late complications—as defined between the discharge and 1-year follow-up—does not differ between the two groups. Almost 40 % of the patients necessitated dilatations because of symptomatic benign stenosis of the gastric tube anastomosis. The median average of dilatations was five per patient. This outcome corresponds with other prospective studies [29, 30].

Interesting is the recovery during the first postoperative year of vocal cord paralysis in patients of the OE group. At 6 weeks after operation, eight patients in the OE group versus one patient in the MIE group suffered from vocal cord paralysis, whereas at 1-year three patients of the OE group had recovered the vocal function. Probably, neuropraxy of the nerve explains this recovery. In accordance with this recovery, scores on the domain ‘talking’ in the EORTC OES18 reveal no more difference between the groups 1 year after surgery.

This study has some limitations. This trial was powered for short-term pulmonary infections and not for mid-term quality of life. However, considering the impaired physical domains of the quality of life which is probably related to post-thoracotomy pain it is likely that this difference with the MIE group would still be present. Other trials, powered for quality of life, are however needed to confirm our results. In addition, the quality of life questionnaires at 1 year was not completed by all patients (82 % completion). Some patients with cancer recurrence did not complete the questionnaires. Given their equal distribution in both groups the influence of the non-responders is small in the final outcome.

In conclusion, this first randomized trial shows that MIE for esophageal cancer is associated with a better mid-term 1-year quality of life compared to open esophagectomy.

## Collaborators

Other members of the TIME-trial research group are: Klinkenbijl JH (Department of Surgery, Academic Medical Centre, Amsterdam, Netherlands), Hollmann MW (Department of Anesthesiology, Academic Medical Centre, Amsterdam, Netherlands), de Lange ES (Department Epidemiology and Biostatistics, Vumc, Amsterdam, Netherlands), Bonjer HJ (Department of Surgery, Vumc, Amsterdam, Netherlands).
